# Senescence‐induced changes in CD4 T cell differentiation can be alleviated by treatment with senolytics

**DOI:** 10.1111/acel.13525

**Published:** 2021-12-27

**Authors:** Erica C. Lorenzo, Blake L. Torrance, Spencer R. Keilich, Iman Al‐Naggar, Andrew Harrison, Ming Xu, Jenna M. Bartley, Laura Haynes

**Affiliations:** ^1^ UConn Center on Aging University of Connecticut School of Medicine Farmington Connecticut USA; ^2^ Department of Immunology University of Connecticut School of Medicine Farmington Connecticut USA; ^3^ Present address: Quantitative Cell Diagnostix Farmington Connecticut USA

**Keywords:** aging, influenza, senescence, senolytics, T cells

## Abstract

Aging and senescence impact CD4 T helper cell (Th) subset differentiation during influenza infection. In the lungs of infected aged mice, there were significantly greater percentages of Th cells expressing the transcription factor FoxP3, indicative of regulatory CD4 T cells (Treg), when compared to young. TGF‐beta levels, which drive FoxP3 expression, were also higher in the bronchoalveolar lavage of aged mice and blocking TGF‐beta reduced the percentage of FoxP3^+^ Th in aged lungs during influenza infection. Since TGF‐beta can be the product of senescent cells, these were targeted by treatment with senolytic drugs. Treatment of aged mice with senolytics prior to influenza infection restored the differentiation of Th cells in those aged mice to a more youthful phenotype with fewer Th cells expressing FoxP3. In addition, treatment with senolytic drugs induced differentiation of aged Th toward a healing Type 2 phenotype, which promotes a return to homeostasis. These results suggest that senescent cells, via production of cytokines such as TGF‐beta, have a significant impact on Th differentiation.

## Graphical abstract

In young mice, CD4 T cells differentiate appropriately in response to influenza infection, generating T helper subsets that participate in the anti‐viral response. This differentiation is aberrant in aged mice, but can be improved when aged mice are treated with senolytics.

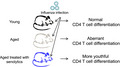

## 1 INTRODUCTION

Aging is one of the greatest risk factors for increased morbidity and mortality following infection. CDC reports indicate that between January 1, 2020−April 28, 2021 a staggering 80% of deaths involving SARS‐CoV‐2, influenza, or pneumonia in the United States were in those ≥65 years of age ([CDC], 2021). There are many factors contributing to this age‐related disparity, however the age‐related changes in the immune system remains an area of heightened clinical concern. These changes manifest in many ways especially in the context of delayed and reduced T cell responses. Both CD4 and CD8 T cells are important for anti‐viral responses, however, CD4 T cells have the unique ability to differentiate into several different specialized subsets important in all stages of viral clearance and recovery.

With advancing age, CD4 T cell differentiation and function declines resulting in skewed subset distribution, diminished cellular and humoral immunity including reduced B cell responses and defective germinal center formation, as well as diminished recruitment of other inflammatory cells to the site of infection (Lefebvre, Masters, et al., 2016). To further highlight the importance of CD4 T cells in influenza infection, it has been demonstrated that depletion of CD4 T cells results in a defect in the recruitment of CD8 T cells to sites of infection and even further, delayed viral clearance (Riberdy et al., 2000). In fact, the roles of CD4 T cells during influenza infection are numerous and include the generation of cytokine‐producing effector populations that can direct the adaptive immune response, generation of T follicular helper subsets that work to help generate high affinity virus neutralizing antibodies and generation of cytotoxic effectors that can kill virus infected cells (Strutt et al., 2013).

Several CD4 T cell subsets have been implicated in the lung response to influenza infection, namely T helper 1 (Th1), T helper 2 (Th2), and regulatory T cell (Treg) subsets. Th1 CD4 T cells produce IFN‐gamma, IL‐2, and TNF‐alpha, and promote activation of CD8 T cells, macrophages, and lung epithelial cells, which supports efficient viral clearance (Strutt et al., 2013). Th2 cells produce IL‐4, IL‐5, IL‐13, IL‐10, and promote recruitment of immune cells to areas of lung epithelial damage, which then promotes healing and a return to homeostasis (Gieseck et al., 2018). Treg cells secrete IL‐10 and TGF‐beta and play an important role in limiting immunopathology, maintaining homeostasis of the lung mucosal environment and dampening of the inflammatory immune response (Okeke & Uzonna, 2019). Th1 cells are key players in viral control and regulation, while Treg and Th2 subsets play key roles in maintaining the balance of inflammation, tissue homeostasis, and healing. Although in young mice and humans there is a balance between these subsets throughout the course of infection, where Th1 cells predominate at early stages of adaptive response to infection and Treg and Th2 subsets predominate at later stages, this balance is disrupted with age and is associated with altered responses (Lefebvre, Lorenzo, et al., 2016; McElhaney et al., 2006). Importantly, an increase in Tregs during influenza infection in aged mice has been appreciated for some time (Williams‐Bey et al., 2011), but the mechanism(s) responsible for this have not yet been elucidated.

CD4 T cells require three key signals in order to clonally expand and differentiate into these specialized subsets. The first signal is the recognition of antigenic peptides through T cell receptor interaction with MHC Class II, which is required for initial activation. The second is further signaling through co‐stimulatory molecules between antigen presenting cells (APCs) and T cells, providing activation or inhibitory signals that direct cell fate and function (Mueller et al., 1989). The third is cytokines and chemokines, which provide additional signals necessary for the migration of newly generated antigen‐specific CD4 T cell subsets to the lung and differentiation to Th subsets which exert effector functions. With aging, chemokine and cytokine signals from the tissue environment are significantly altered and delayed, which support skewed CD4 T cell differentiation, migration, and expansion (Lefebvre, Lorenzo, et al., 2016; Lefebvre et al., 2012). Here, we hypothesize that senescent cells and their secretomes are one of the main contributors to the altered aged tissue environment.

Cellular senescence is characterized by irreversible growth arrest and plays a role throughout various stages of life. In development, cellular senescence is critical for proper tissue and organ formation, while after birth and throughout adulthood, it is key in protecting against potentially oncogenic insults by preventing cells from dividing with DNA and or mitochondrial damage. Senescent cells in adulthood are unable to go through apoptotic pathways of self‐elimination and rely on phagocytic cells of the immune system for clearance (Di Micco et al., 2021). Most importantly, the number of senescent cells increases with chronological aging, as accumulation surpasses their rate of elimination (Di Micco et al., 2021). Although senescence leads to irreversible cell cycle arrest, senescent cells remain metabolically active and display many cellular changes. Senescent cells become resistant to apoptosis, have protein aggregation in the endoplasmic reticulum, and marked dysfunction in mitochondria and lysosomes (Rayess et al., 2012).

Senescent cells also express a senescence associated secretory phenotype (SASP), which has been described as the ability to secrete multiple growth factors, cytokines and chemokines, which attract immune cells such as macrophages and natural killer cells to aid in their elimination (Coppe et al., 2008). It has been shown, however, that if senescent cells are not effectively cleared, they can accumulate and chronically secrete inflammatory cytokines, such as IL‐6 and IL‐8, which can negatively affect self‐elimination (Coppe et al., 2008). Thus, the accrual of senescent cells with aging has further deleterious effects on neighboring cells and tissues and contributes to the aged microenvironment.

Senescent cells have been implicated in many age‐related conditions and diseases and are thought to be one of the main drivers of aging. A number of these conditions have been extensively described by us and others in recent studies demonstrating a significant impact of senescence including: physical dysfunction (Xu et al., 2015), osteoporosis (Farr et al., 2017), adipose tissue dysfunction (Xu et al., 2015), osteoarthritis (Xu et al., 2017), cardiac dysfunction (Baker et al., 2016), kidney dysfunction (Baker et al., 2011), atherosclerosis (Childs et al., 2016), liver steatosis (Ogrodnik et al., 2017), pulmonary fibrosis (Schafer et al., 2017), stem cell dysfunction (Xu et al., 2015) and lifespan reduction (Baker et al., 2016). Taken together, it is clear that senescence plays a large role in poor health and disease progression in older adults, and that treatments to mitigate these effects are critical to improving health and quality of life.

Importantly, senolytics, which are drugs that can specifically target senescent cells, are now being developed and offer the promise of delaying many different age‐related conditions (Niedernhofer & Robbins, 2018). A combination of two senolytic drugs, dasatinib (D), a pan‐tyrosine kinase inhibitor, and quercetin (Q), a plant flavonoid, has been shown to eliminate senescent cells and alleviate several age‐related diseases (Xu et al., 2018). Recently, a pilot study in a small set of idiopathic pulmonary fibrosis (IPF) patients aged ≥50 years determined that intermittent administration of a D+Q cocktail resulted in a significant improvement in physical function (Justice et al., 2019). These results also confirm observations in aged mice (Xu et al., 2018) and IPF mice (Schafer et al., 2017), indicating that mouse models are valid for human aging and senescence research. In this current study, we examine the effects of D+Q treatment on CD4 T cell differentiation *in vivo* in an aging mouse model. Importantly, our results show that targeting senescent cells with D+Q can alter the pattern of differentiation of CD4 T cells in aged mice to more closely resemble that found in younger mice. While many studies have demonstrated the impact of senescence on age‐related disease progression and physical function, only a few have addressed how this impacts immune function (Camell et al., 2021; Palacio et al., 2019). Importantly, ours is the first study to suggest a relationship between the senescent environment and its impact on the differentiation of CD4 T cells in response to an infectious challenge.

## 2 RESULTS

### 2.1 Aging impacts CD4 T cell differentiation during influenza infection

**FIGURE 1:**
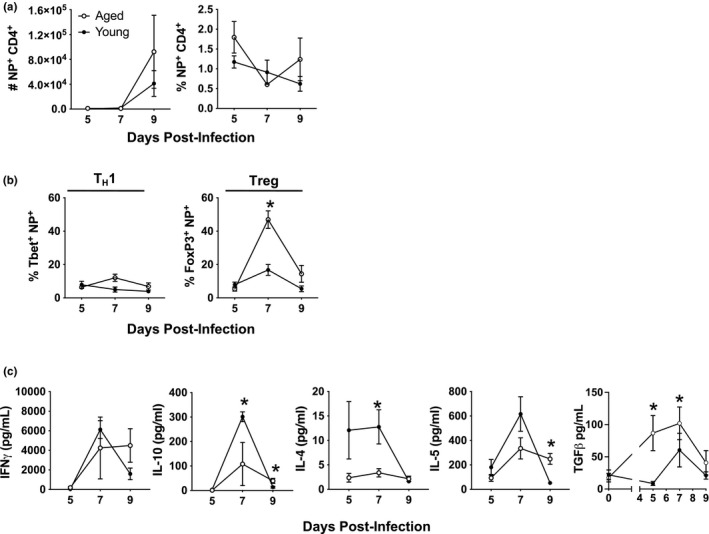
Higher levels of FoxP3 expressing CD4 T cells in the lungs of influenza infected aged mice. Young (6–8 weeks) and aged (18–20 months) C57BL/6 mice were infected with a sublethal dose of influenza and on days 5, 7, and 9 post‐infection, lungs were harvested. NP‐specific CD4 T cells were enumerated within the total CD4 T cell population with a NP_311‐325_/IA^b^ MHC class II tetramer. (a) Shows the number and percentage of NP^+^ flu‐specific cells within the total CD4 T cell compartment in the lung at each time point. (b) The percentage of NP^+^ flu‐specific cells within the CD4 T cell compartment was assessed for expression of Th subset‐specific transcription factors Tbet (for Th1) and FoxP3 (for Treg) by flow cytometry. (c) Bronchoalveolar lavage fluid (BAL) was also harvested from the young and aged mice. IFNγ, IL‐10, IL‐4, and IL‐5 in the BAL were measured by multiplex assay and free active TGF‐beta was measured by ELISA. Data shown are combined from at least four independent experiments with a total of 5–15 mice per group. Statistical significance was calculated using the Mann–Whitney *U* test with Holm–Sidak post hoc corrections for multiple comparisons. The data is presented as means ± standard error of the mean (SEM); *p ≤ 0.05

In order to examine how aging and senescence impact CD4 T cell differentiation we have used our well‐developed model of sublethal influenza infection (Lefebvre, Masters, et al., 2016). During influenza infection, CD4 T cells differentiate into subsets such as Th1 to help clear the virus and Treg to control immunopathology. These subsets are characterized by expression of the transcription factors Tbet and FoxP3, respectively (Abbas et al., 1996; Josefowicz & Rudensky, 2009; Tripathi & Lahesmaa, 2014). The CD4 subsets in the lungs of young and aged infected mice were examined using MHC Class II tetramers to identify influenza nucleoprotein (NP)‐specific CD4 T cells. The total numbers and percentages of NP‐specific CD4 T cells in the lungs were similar in young and aged groups (Figure 1a). The differentiation of responding CD4 T cells into Th subsets in each of these groups was then examined by determining transcription factor expression using a flow cytometric approach (Figure S1). Figure 1b shows that in the lungs there was no difference in the percentage of Tbet^+^ Th1 NP‐specific CD4 T cells in young and aged mice. In contrast, there was a significantly higher percentage of FoxP3^+^ Tregs in aged lungs when compared to young at days 7 and 9 post‐infection.

Bronchoalveolar lavage fluid (BAL) was also collected from the lungs of these young and aged mice and cytokines were quantified. While there was no significant difference in IFN‐gamma levels, there were significantly higher levels of the Type 2 cytokines IL‐10 and IL‐4 in BAL in young mice when compared to aged at day 7 (Figure 1c). Interestingly, the Type 2 cytokines IL‐5 and IL‐10 are significantly higher in the BAL from aged groups at day 9. This is noteworthy since Type 2 cytokines are important for resolution of an immune response and a return to homeostasis following infection (Allen & Wynn, 2011). Additionally, since FoxP3 expression in CD4 T cells is driven by TGF‐beta (Xu et al., 2010), we quantified this and observed that while there was no difference in TGF‐beta in the BAL in uninfected young and aged mice, significantly higher levels of TGF‐beta were present in aged groups on days 5 and 7 post‐infection when compared to young mice.

### 2.2 TGF‐beta drives disproportionate Treg differentiation

TGF‐beta is a pleotropic cytokine secreted by many cell types in the lung including epithelial cells, other fibroblastic‐type cells, and immune cells such as macrophages and it plays a role in the reinforcement of senescent cell phenotypes (Rapisarda et al., 2017). Not only does exogenous TGF‐beta impact senescent cells, but endogenous TGF‐beta production has also been shown to be upregulated in human fibroblasts that have become senescent (Rapisarda et al., 2017) and TGF‐beta can be a component of the SASP (Borodkina et al., 2018; Hubackova et al., 2012). This suggests that TGF‐beta from senescent cells, which are more plentiful in aged mice (Burd et al., 2013), may be an important contributing factor to the aged tissue microenvironment that will further influence CD4 T cell polarization, especially to a more regulatory phenotype.

**FIGURE 2:**
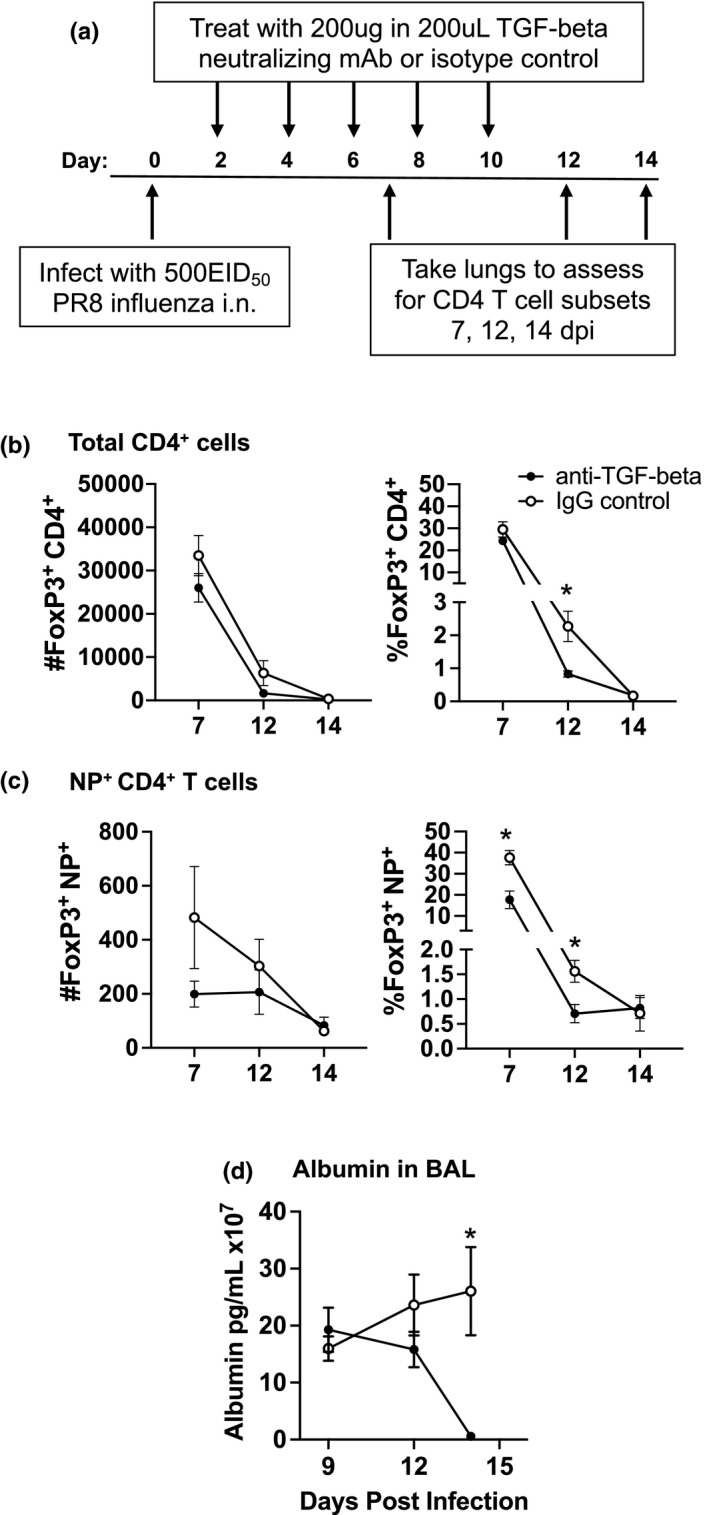
Neutralization of TGF‐beta in aged mice reduced the number of FoxP3^+^ cells in the lungs. (a) Aged mice were treated with anti‐TGF‐beta neutralizing monoclonal antibody (BioXcell, clone: 1D11.16.8) or IgG isotype control (IgG) on 2, 4, 6, 8, and 10 days following flu infection. Mice were sacrificed on days 7, 12 and 14 and the lungs were harvested for flow cytometry to determine the effect of TGF‐beta neutralization on the CD4^+^FoxP3^+^ Treg subset in (b) the total CD4 T cell compartment and (c) the NP‐specific CD4 T cell compartment using an NP_311‐325_/IA^b^ MHC class II tetramer. Each symbol represents a single animal. (d) In a separate experiment, anti‐TGF‐beta treated mice were sacrificed on days 9, 12 and 15 post‐infection and the amount of albumin in the Bronchoalveolar lavage fluid (BAL) was assessed by ELISA. For all, statistical significance was calculated using the Mann–Whitney *U* test with Holm–Sidak post hoc corrections for multiple comparisons comparing anti‐TGF‐beta treated and IgG isotype control groups at each time point. The data is presented as mean ± standard error of the mean (SEM) with *n* = 4–8 animals/group. *p ≤ 0.05

Based on the results shown in Figure 1, we treated aged mice with neutralizing anti‐TGF‐beta monoclonal antibody following infection in order to determine if the TGF‐beta in the aged environment impacted FoxP3 expression in lung CD4 T cells (Figure 2a). Treatment reduced the percentage of FoxP3 expressing Th cells in both the total lung CD4 T cell population (Figure 2b; Figure S2a) and the NP‐specific CD4 T cell population (Figure 2c; Figure S2b). Unexpectedly however, anti‐TGF‐beta treatment also dramatically decreased the level of albumin found in the BAL of the infected aged mice (Figure 2d). These results show that not only does the high level of TGF‐beta in the lungs of aged mice drive the disproportionate increase in FoxP3 expression and Treg differentiation, but also that one of the consequences of greater numbers of Tregs is increased lung damage, since the presence of albumin in the BAL is associated with lung damage (Bhan et al., 2015). While these results can implicate TGF‐beta in altered CD4 Th subset differentiation over the course of infection, it does not determine whether the senescent environment or senescent cells themselves are the main drivers of this difference with age. As a result, we then aimed to study the contribution of pre‐existing senescent cells in this model.

### 2.3 Senescent cells contribute to age‐related changes in CD4 T cell differentiation

**FIGURE 3:**
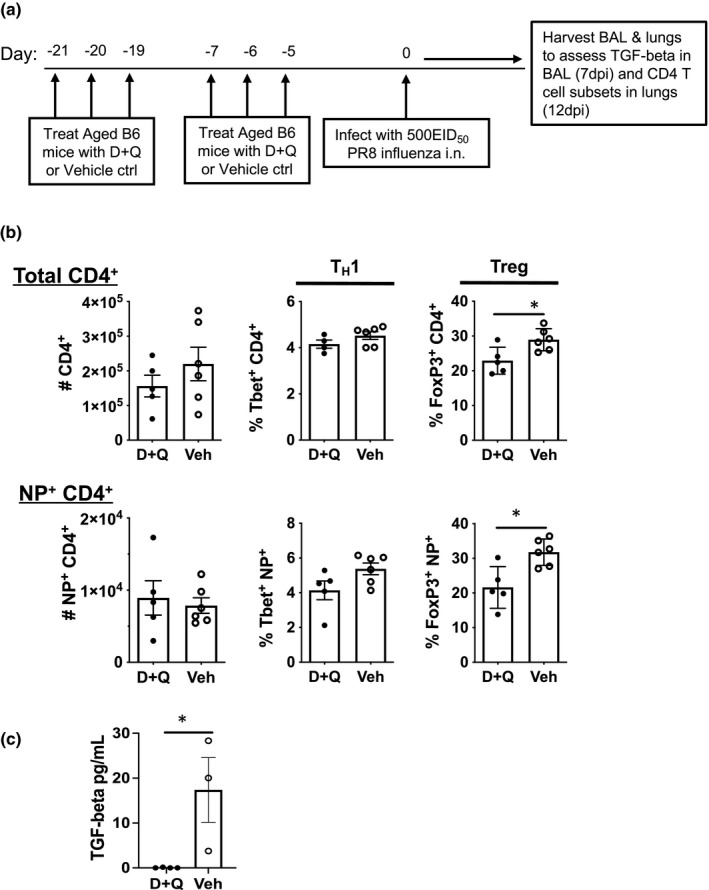
Senolytic drug combination D+Q induces reduction in FoxP3^+^ CD4 T cell subsets in the lung and TGF‐beta in the Bronchoalveolar lavage fluid (BAL). (a) Aged mice were treated with D+Q (5 mg/kg/day Dasatinib and 50 mg/kg/day Quercetin) by oral gavage prior to flu infection as described in the Experimental Procedures section. (b) Mice were sacrificed on day 12 post‐infection and lungs were harvested for flow cytometry to determine the effect of D+Q treatment on transcription factor expression by total CD4^+^ and NP^+^CD4^+^ cells. (c) Free‐active TGF‐beta was measured in the BAL and analyzed by ELISA on day 7 post‐infection. Data shown is from one experiment with a total of 5 mice per D+Q treated group and 6 mice per vehicle treated group. Statistical significance was calculated using the Mann–Whitney *U* test comparing D+Q and Veh treated groups. The data is presented as means ± standard error of the mean (SEM). *p ≤ 0.05

Aged mice have more senescent cells when compared to young mice (Xu et al., 2018) and we hypothesized that these senescent cells or the senescent environment might be contributing to the aging environment that is driving the increased expression of FoxP3 and differentiation to Tregs. Importantly for this study, TGF‐beta can be generated by senescent cells as a component of the SASP (Borodkina et al., 2018; Hoare et al., 2016). To more directly examine the impact of senescent cells on Th subsets in aged mice, senescent cells were targeted by administration of the senolytic drug cocktail dasatinib and quercetin (D+Q) (Xu et al., 2018) prior to influenza infection of aged mice (Figure 3a). We chose this approach since it has been shown to be very effective at targeting senescent cells in the lung (Schafer et al., 2017). Since the treatment regimen ends 5 days prior to infection and these drugs have a very short half‐life (Christopher et al., 2008), the effect of these senolytic drugs should be restricted to existing senescent cells and should not impact the future anti‐viral immune response.

Treatment of aged mice with D+Q does not have an impact on Th1 differentiation in the total lung CD4 T cell population or the NP‐specific populations, but it does impact Treg differentiation. In the D+Q treated group, the percentage of FoxP3 expressing Treg cells is significantly reduced on day 12 post‐infection as compared to vehicle treated (Figure 3b; Figure S3). We also examined CD8 T cells in the lungs of these mice and found that D+Q treatment had no impact on cell numbers, percentages of NP‐specific cells or effector subsets (as previously defined, Lefrancois & Obar, 2010; Figure S4). Importantly, we found that the concentration of TGF‐beta in the BAL is significantly reduced (Figure 3C) in D+Q treated aged mice when compared to the vehicle control group. This suggests that previously existing senescent cells might be contributing to TGF‐beta production and have an impact on Treg differentiation following infection, even weeks following senolytic treatment.

### 2.4 Impact of D+Q treatment on BAL cytokines

**FIGURE 4:**
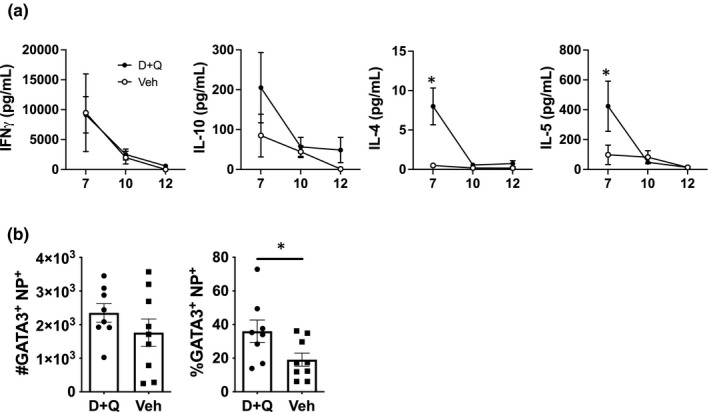
D+Q treatment is associated with the production of Type 2 cytokines in aged lungs. (a) Prior to flu infection, aged mice were treated with D+Q or vehicle as in Figure 3. On days 7, 10, 12, and 14 post‐infection, Bronchoalveolar lavage fluid was harvested and IFNγ, IL‐10, IL‐4, and IL‐5 were measured by multiplex assay. Data shown is from one experiment with a total of 5 mice per D+Q treated group and 6 mice per vehicle treated group (b) Aged mice were treated with D+Q or vehicle before flu infection. On day 12 post‐infection, lung tissue was harvested and NP‐specific CD4 T cells were enumerated with a NP_311‐325_/IA^b^ MHC class II tetramer. The number and percentage of NP^+^ flu‐specific cells within the total CD4 T cell compartment were assessed for expression of T_H_2 subset‐specific transcription factor GATA3 by flow cytometry. Data shown is from one experiment with a total of 8–9 mice per group. Statistical significance was calculated using the Mann–Whitney *U* test with Holm–Sidak post hoc corrections for multiple comparisons comparing D+Q and vehicle treated groups at each time point. All data are presented as means ± standard error of the mean (SEM). *p ≤ 0.05

We next assessed cytokines in the BAL from aged mice treated with D+Q or vehicle in order to determine if senolytics could impact overall cytokine production. There was no difference in IFN‐gamma in the two groups, but the D+Q treated aged mice had significantly higher levels of IL‐4 and IL‐5 when compared to the vehicle treated group (Figure 4a). This pattern of cytokine production in the D+Q treated group is somewhat similar to what was found in the BAL of young mice (Figure 1c). We also found that aged mice treated with D+Q exhibited a higher percentage of GATA3 expressing influenza NP‐specific CD4 T cells (Figure 4b; Figure S5). Taken together, these results indicate that D+Q treatment seems to be promoting a more balanced CD4 differentiation pattern in aged mice, including the inclusion of a more Th2‐like phenotype (Tripathi & Lahesmaa, 2014). This result also indicates that it is likely that the presence of senescent cells negatively impacts the CD4 T cell differentiation program in aged mice, which, in turn, could then impact the initiation of healing and return to homeostasis following viral clearance, which is mediated by Th2 cells (Allen & Wynn, 2011).

### 2.5 The senescent environment impacts differentiation of young CD4 T cells

**FIGURE 5:**
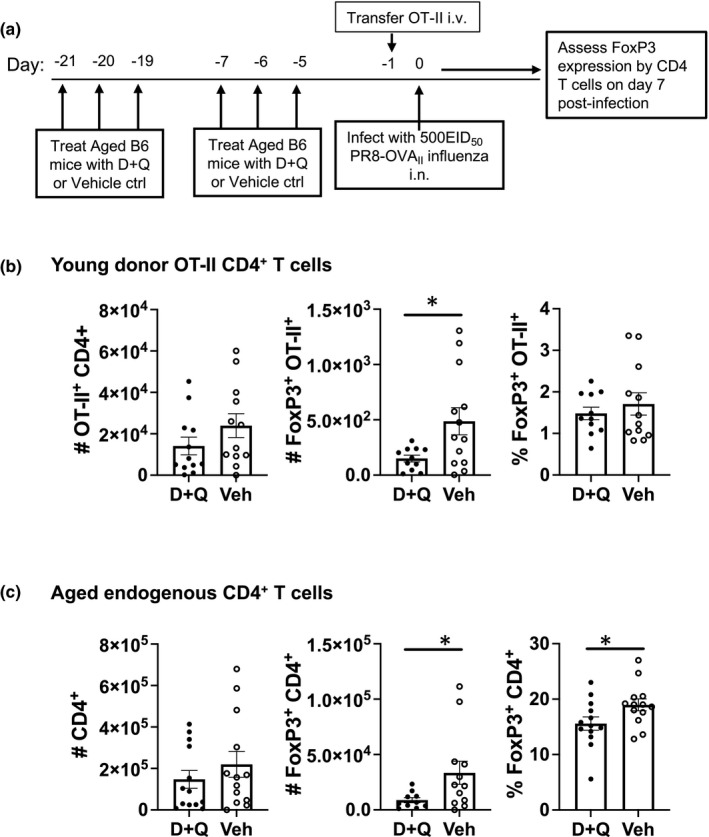
The senescent environment drives both endogenous aged CD4 and transferred young CD4 T cells to express FoxP3. (a) Young OT‐II CD4^+^ T cells (5 × 10^5^) were transferred i.v. into aged C57BL/6 mice hosts that were previously treated with D+Q or vehicle control (as in Figure 3) and then subsequently infected with PR8‐OVA_II_ influenza. On day 7 post‐infection, (b) the young OT‐II donor CD4^+^ T cells and (c) the aged host endogenous CD4^+^ T cell populations were isolated from the mediastinal lymph node and were quantified for expression of FoxP3. Data shown is combined from two independent experiments with a total of 10–15 mice per group. Statistical significance was calculated by Student's *t* test comparing D+Q and Veh treated groups. The data is presented as means ± standard error of the mean (SEM). *p ≤ 0.05

It is possible that the results observed in Figure 1 may be influenced by an increased predisposition of aged influenza‐specific CD4 T cells to express Foxp3 and differentiate toward a Treg phenotype when compared to young CD4 T cells. In order to address this point, aged mice were treated with D+Q or vehicle control and then young OT‐II ovalbumin (OVA) specific CD4 T cells were transferred into these aged hosts (Figure 5a). All hosts were then infected with an influenza virus expressing the OVA peptide that is recognized by OT‐II CD4 T cells (PR8‐OVA_II_) and the phenotype of both the transferred and endogenous CD4 T cells in the mediastinal lymph node was examined 7 days later. Fewer donor OT‐II CD4 T cells expressing FoxP3 were recovered from D+Q treated hosts when compared to vehicle treated hosts (Figure 5b). The endogenous aged CD4 T cells exhibited a similar trend with fewer of them expressing FoxP3 (Figure 5c). Taken together, these results indicate that targeting senescent cells with senolytic drugs reduces the differentiation of responding CD4 T cells into a FoxP3‐expressing Treg phenotype, regardless of whether they originated in a young or aged mouse.

## 3 DISCUSSION

It is well established that the immune response to influenza infection is significantly impacted by aging. We have shown previously that: (1) clearance of influenza virus is delayed in aged mice; (2) inflammatory cytokines (G‐CSF, IL‐6, IL‐1‐alpha) linger longer in the BAL from aged mice; (3) albumin stays elevated longer in the BAL from aged mice; and (4) there are age‐related perturbations in Th subset differentiation as defined by cell surface markers (Lefebvre, Lorenzo, et al., 2016). The relationship between these four points remains to be delineated and will be the subject of intense research over the coming years as we employ new approaches to investigate aging complications. While there are obvious intrinsic age‐related changes in CD4 T cells (Eaton et al., 2004; Haynes et al., 1999), previous studies from our laboratory have also shown that the aged microenvironment has a distinct impact on the response of CD4 T cells when compared to a young environment (Lefebvre, Masters, et al., 2012; Lefebvre et al., 2012; Masters et al., 2019). In the aged environment, chemokine expression, cellular organization, and organ architecture are all dysregulated in secondary lymphoid organs, which can then impact both the initial priming and subsequent differentiation of responding CD4 T cells. Importantly, we have demonstrated that significantly more T follicular regulatory cells (T_FR_, Bcl6^+^FoxP3^+^) develop in aged mice when compared to young during influenza infection (Lefebvre, Masters, et al., 2016). Additionally, polyclonal young CD4 T cells transferred into aged mice were more likely to express a T_FR_ phenotype when compared to those transferred into young mice and this was accompanied by higher concentrations of TGF‐beta in the spleens of the aged hosts (Lefebvre, Masters, et al., 2016). Taken together, these results indicate that the aged TGF‐beta‐rich environment favors Treg differentiation, but the mechanisms responsible for this were not apparent at the time.

In this current study, we have expanded on the examination of Th subset differentiation, now using lineage‐specific transcription factors to define Th subsets. Importantly, this approach revealed that while there was no significant difference in the percent of influenza NP‐specific CD4 T cells expressing Tbet in young and aged lungs, there were significantly more FoxP3‐expressing CD4 T cells in aged lungs. These FoxP3^+^ Tregs have the potential to down regulate effector mechanisms that are important for viral clearance, including the generation of anti‐viral CD8 T cells, which could, in turn, delay viral clearance.

While these results are both novel and interesting, there are notable limitations with our studies and their interpretations. Firstly, although the half‐life of D+Q is short and it is cleared from the mice within 24 h (Christopher et al., 2008; Graefe et al., 2001), it is possible that D+Q might have off‐target effects on T cells. In our studies, we have tried to mitigate off‐target effects by including a 5‐day wash‐out period during which the drug is rapidly metabolized and excreted. In addition, T cell proliferation and differentiation is not seen in our model until about days 5 or 7 post‐infection. Therefore, although we did not examine the effects of D+Q on T cells directly, there is little evidence to support that T cells will be affected by a D+Q treatment 10–12 days earlier. More critically, because the in vivo study of senescent cells is still a nascent field, it is very difficult to definitively link the use of senolytics to targeting specific cell types in a given tissue, e.g., the lungs. Furthermore, there is no single reliable biomarker for senescent cells that would allow us to track the overall burden before senolytic treatment and during infection in the same mouse. This greatly complicates our ability to clearly demonstrate that senolytic treatment decreases senescent cell burden in the lung that then goes on to confer beneficial outcomes in CD4 T cell differentiation in the same individual mouse. It is also difficult, for these same reasons, to determine whether senescent cells are the sole producers of TGF‐beta during a typical aged response to flu infection. As the field develops and more reliable biomarkers are identified, these details will be more clearly elucidated.

Thus, there remain open questions that require further investigation in this model. Some of the most important questions that remain to be answered are: (1) Which cells are becoming senescent? (2) Does the age‐related increase in Tregs have an impact on the primary response to influenza infection (including viral clearance) in our model as other investigators have reported (Williams‐Bey et al., 2011)? (3) Do these Tregs have an impact on the development of protective immune memory in aged mice? (4) Do Tregs generated in aged mice have suppressive activity that is similar to those in young mice? (5) Does TGF‐beta in the SASP drive de novo differentiation of inducible Tregs or does it promote the survival and proliferation of existing Tregs? These points are all important especially in light of a recent study demonstrating that treatment of aging mice with senolytics can improve survival and antibody production, both indicative of a robust adaptive immune function, in response to a viral infection (Camell et al., 2021). The results of our study suggest that these improvements in immunity could be the result of changes in CD4 T cell differentiation in the treated aged mice.

Important for our studies is the fact that there is an accumulation of senescent cells in the aging lung environment (Campisi, 2016) and thus, this could impact the immune response to influenza infection. In the lung, fibroblasts, smooth muscle cells, and alveolar epithelial cells can become senescent and may contribute to lung disease and could also contribute to a senescent environment that impacts subsequent adaptive immune responses. Importantly, some SASP factors are also central players in CD4 T cell homeostasis, stimulation, and differentiation including IL‐1alpha, IL‐6, IL‐7 and TGF‐beta (Coppe et al., 2008; Hubackova et al., 2012) and have the potential to impact adaptive immune responses. In addition, it has been shown that senescent cells exhibit a hyper‐inflammatory response to pathogen‐associated molecular patterns (PAMPS), which could account for some of these age‐related changes in the cytokine milieu (Camell et al., 2021). Our results demonstrate that senolytic drugs can impact CD4 T cells, most likely by modulating the microenvironment, which then can influence T cell differentiation during the response to influenza infection. These promising results suggest that aspects of diminished immune responses with age are in fact treatable and future studies may be able to design such therapeutics for older adults to prevent influenza and other infectious disease mortality.

## 4 EXPERIMENTAL PROCEDURES

### 4.1 Mice

Young (2–3 months) male C57BL/6 mice were purchased from Jackson Laboratories or obtained from the National Institute on Aging. Aged (19–22 months) male C57BL/6 mice were obtained from the National Institute on Aging rodent colony. OT‐II. Thy1.1 mice were bred at UConn Health. All mice were housed in a climate‐controlled environment and fed standard rodent chow and water ad libitum until use. All mice were cared for in accordance with the recommendations in the Guide for the Care and use of Laboratory Animals of the National Institutes of Health. All procedures were approved by the UConn Health IACUC.

### 4.2 Viral infection

Mice were anesthetized with isoflurane and intranasally inoculated with 500 EID_50_ of the H1N1 influenza virus A/PR/8/34 (PR8) or PR8 expressing an MHC Class II‐restricted ovalbumin peptide (PR8‐OVA_II_) in 50 μl PBS. After infection, mice were weighed daily to monitor infection progression. Recumbent mice and mice that had lost more than 30% body weight were considered moribund and euthanized. All mice underwent gross pathological examination at time of sacrifice and animals with obvious pathology were excluded from the study.

### 4.3 Adoptive transfer of CD4 T cells

OT‐II. Thy1.1 CD4 T cells were isolated from the spleens of young (2–4 months old) transgenic mice using Miltenyi negative selection kits and 5 × 10^5^ cells were transferred intravenously into aged mice that had been previously treated with D+Q or vehicle. On the next day, hosts were infected with PR8‐OVA_II_ and then sacrificed on day 7 post‐infection. For analysis, donor OT‐II cells were identified by Thy1.1 staining.

### 4.4 Antibody treatments

Anti‐TGF‐beta neutralizing antibody clone 1D11.16.8 or IgG isotype matched control clone MOPC‐21 (both purchased from BioXell) was delivered intraperitoneally to mice (200ug in 200 μl PBS) on days 2, 4, 6, 8, and 10 post‐influenza infection. This dose of anti‐TGF‐beta antibody has been previously shown to reduce TGF‐beta levels in vivo (Greco et al., 2015).

### 4.5 D+Q treatments

Mice were treated by oral gavage with either 5 mg/kg/day dasatinib and 50 mg/kg/day quercetin or vehicle control administered for three consecutive days in two treatments one week apart. Dasatinib and quercetin were dissolved in vehicle containing 10% ethanol, 30% polyethylene glycol 400, and 60% Phosal 50 PG at a concentration of 2 mg/ml and 20 mg/ml, respectively. Solutions were left to sit rotating gently at room temperature for several hours to dissolve. After the treatment regimen was completed, mice were allowed to rest for 5 days prior to use in experiments.

### 4.6 Multiplex quantification of cytokines

Bronchoalveolar lavage fluid (BAL) was collected by flushing lungs with 1mL saline postmortem. Supernatant was collected after centrifugation and assayed for cytokine and chemokine content using the Luminex Mouse Cytokine/Chemokine 32‐plex panel or 25‐plex panel (EMD Millipore).

### 4.7 Active TGF‐beta1 ELISA

TGF‐beta1 was measured in the BAL supernatants using the LEGEND MAXTM Free Active TGF‐beta1 ELISA kit (BioLegend). Samples were run in duplicate according to manufacturer's instructions.

## 5 ALBUMIN QUANTIFICATION

The albumin content in BAL supernatant was determined by ELISA (Bethyl Laboratories) according to manufacturer's instructions.

### 5.1 Flow cytometry

Single cell suspensions were generated from the lungs or lymph nodes. Cells were incubated with Fc block (anti‐CD16/32) for 15 min on ice followed by staining with a NP_311‐325_ IA^b^ MHC Class II tetramer or NP_366‐374_ H‐2D^b^ MHC Class I tetramer (generated by the NIH Tetramer Core Facility) for 1 h at room temperature at a dilution of 1:50 to ensure optimal staining. For CD8 effector subset analysis, cells were subsequently stained for expression of CD8, KLRG1 and CD127. In transcription factor studies, anti‐CD4 antibody was used in combination with the NP MHC Class II Tetramer and transcription factor antibodies: Tbet (BioLegend), FoxP3 (BioLegend), GATA3 (BioLegend). For intracellular transcription factor detection, cells were fixed and permeabilized using the Foxp3/Transcription Factor Staining Buffer Set (eBioscience) according to the manufacturer's instructions.

### 5.2 Statistics

Differences between experimental groups were analyzed using Student's *t* Test or the Mann–Whitney *U* test with Holm–Sidak post hoc corrections for multiple comparisons when necessary. Statistical analyses were performed with Prism 8 software (GraphPad Software Inc.) or SPSS Software (IBM). Differences were considered significant at *p* < 0.05.

## CONFLICT OF INTEREST

LH serves as a paid consultant for Spring Discovery. MX has a financial interest related to this research: patents on senolytic drugs (including PCT/US2016/041646, filed at the US Patent Office) are held by Mayo Clinic. Other authors declare no conflicts.

## AUTHOR CONTRIBUTIONS

ECL was involved with experimental design, project funding, execution of the experiments, analysis of the results, and assisted with writing of this manuscript; BLT assisted with execution of the experiments, analysis of the results, and assisted with writing of this manuscript; AH helped carry out the experiments; IA‐N, MX, JMB and LH helped with experimental design, interpretation of the results and with the writing of this manuscript; LH obtained the funding that supported this study.

## Supporting information

Fig S1

Fig S2

Fig S3

Fig S4

Fig S5

## Data Availability

The data that support the findings of this study are available from the corresponding author upon reasonable request.
