# Supplementary material for: Senescence‐induced changes in CD4 T cell differentiation can be alleviated by treatment with senolytics
Source: Aging Cell. 2021 Dec 27;21(1):e13525. doi: 10.1111/acel.13525 (PMC8761018; doi:10.1111/acel.13525)
Supplement: Supplementary file 1 — Fig S1 [file ACEL-21-e13525-s003.pdf]

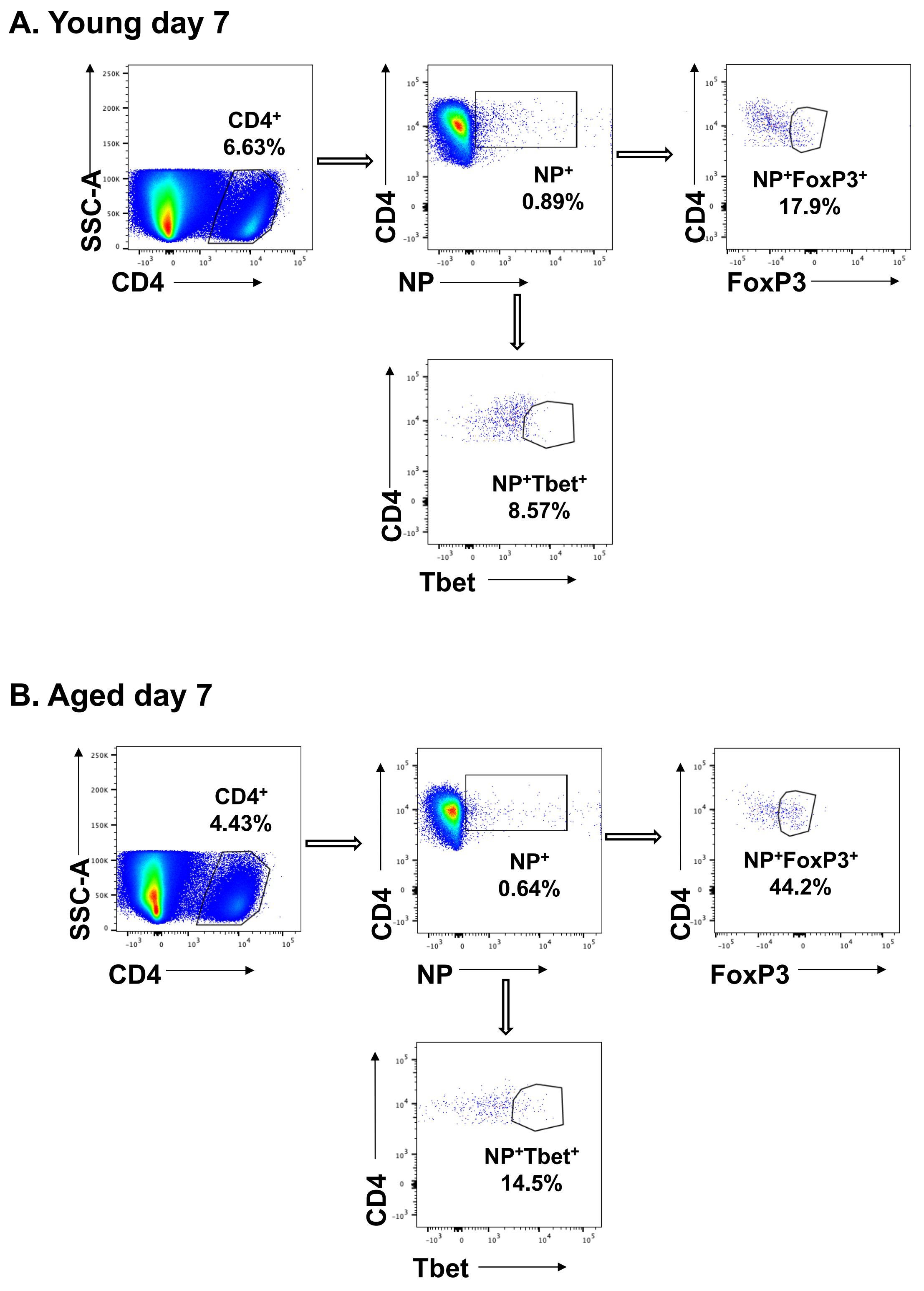

Supplemental figure 1. Flow cytometric analysis of CD4 T cells from young and aged lungs. Shows the gating strategy from the day 7 time point in Figure 1B. Concatenated dot plots indicate the percent positive for FoxP3 and Tbet expression.

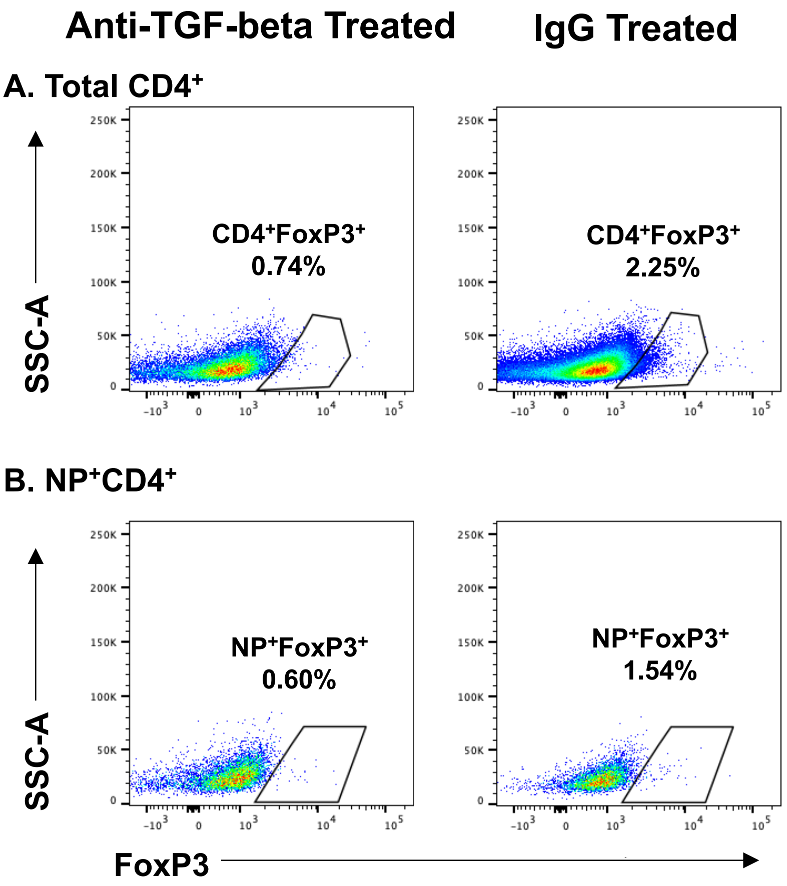

Supplemental figure 2. Flow cytometric analysis of CD4 T cells from anti-TGF-beta treated aged mice. Shows concatenated dot plots indicating the percent positive for FoxP3 and Tbet expression at the day 12 time point in Figure 2B (A. total CD4) and 2C (B. NP-specific CD4).

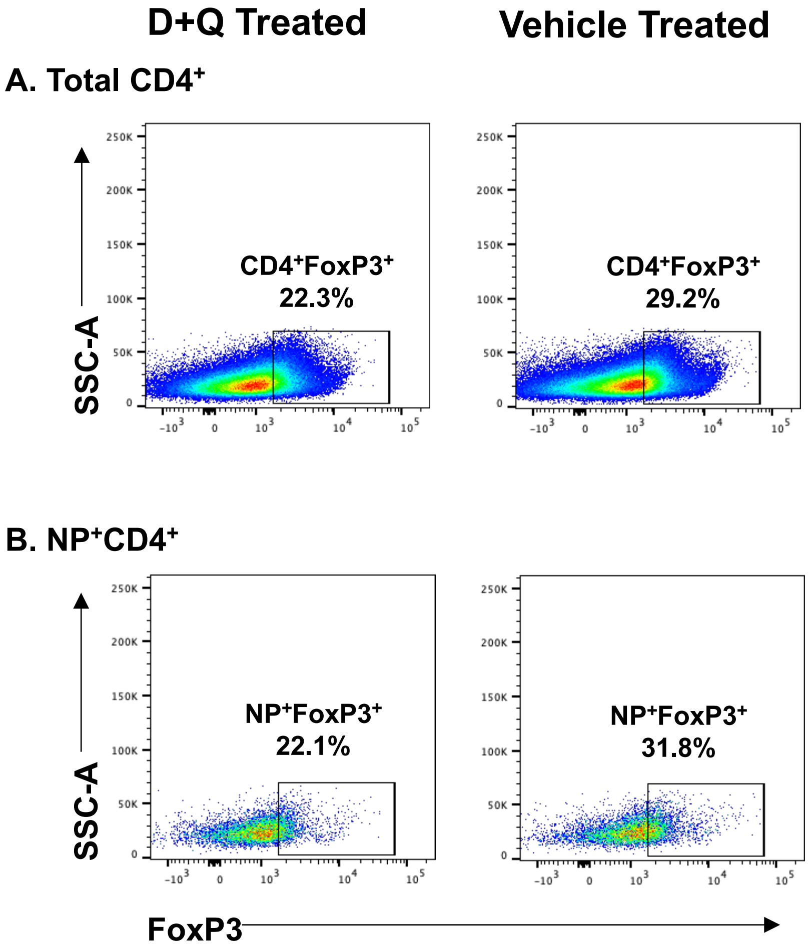

Supplemental figure 3. Flow cytometric analysis of CD4 T cells from D+Q treated aged mice. Shows concatenated dot plots indicating the percent positive for FoxP3 expression in: A. Total CD4 population and B. NP-specific CD4 population from Figure 3B.

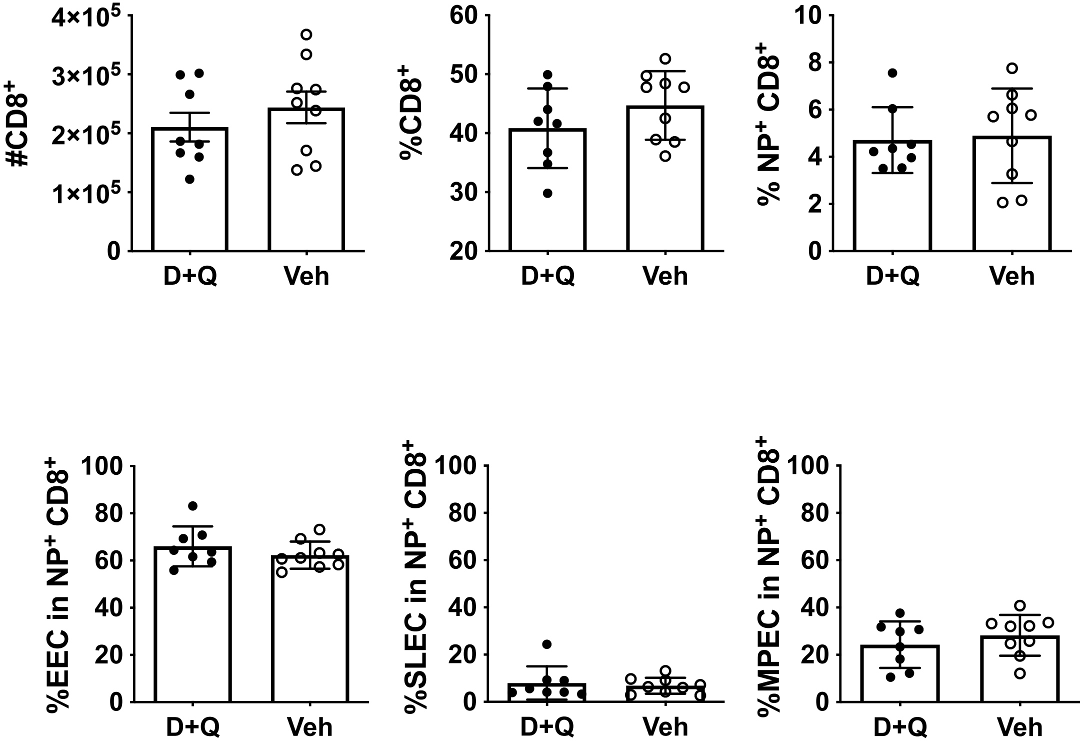

Supplemental figure 4. The impact of D+Q treatment on CD8 T cells in the lung during influenza infection. The experimental setup is shown in Figure 3. On day 12 post-infection, lymphocytes were recovered from the lungs. Flow cytometric analysis was used to identify the total CD8 T cell population and influenza NP-specific CD8 T cells (using H-2D^{b} influenza A NP$_{366-374}$ tetramer). CD8 T cell subsets were also identified: Early Effector Cells (EEC) KLRG-1^{lo} CD127^{lo}; Short Lived Effector Cells (SLEC) KLRG-1^{hi} CD127^{lo}; Memory Precursor Effector Cells (MPEC) KLRG-1^{lo} CD127^{hi}.

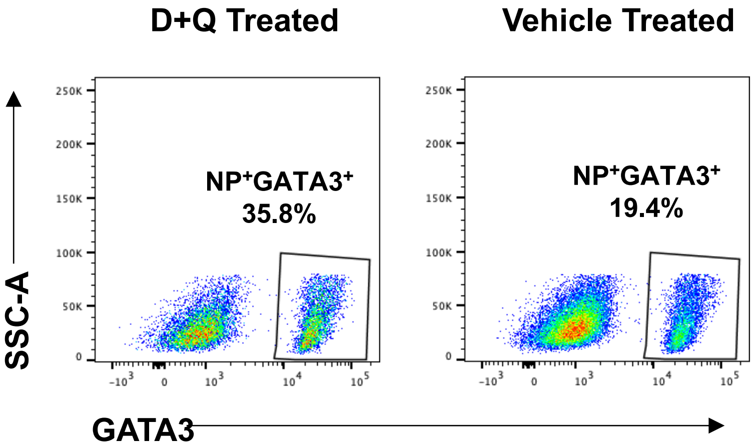

Supplemental figure 5. Flow cytometric analysis of NP-specific CD4 T cells from D+Q treated aged mice. Shows concatenated dot plots indicating the percent positive for GATA3 expression in the NP-specific CD4 population from Figure 4.
