# Supplementary material for: Senescence‐induced changes in CD4 T cell differentiation can be alleviated by treatment with senolytics
Source: Aging Cell. 2021 Dec 27;21(1):e13525. doi: 10.1111/acel.13525 (PMC8761018; doi:10.1111/acel.13525)
Supplement: Supplementary file 2 — Fig S2 [file ACEL-21-e13525-s001.pdf]

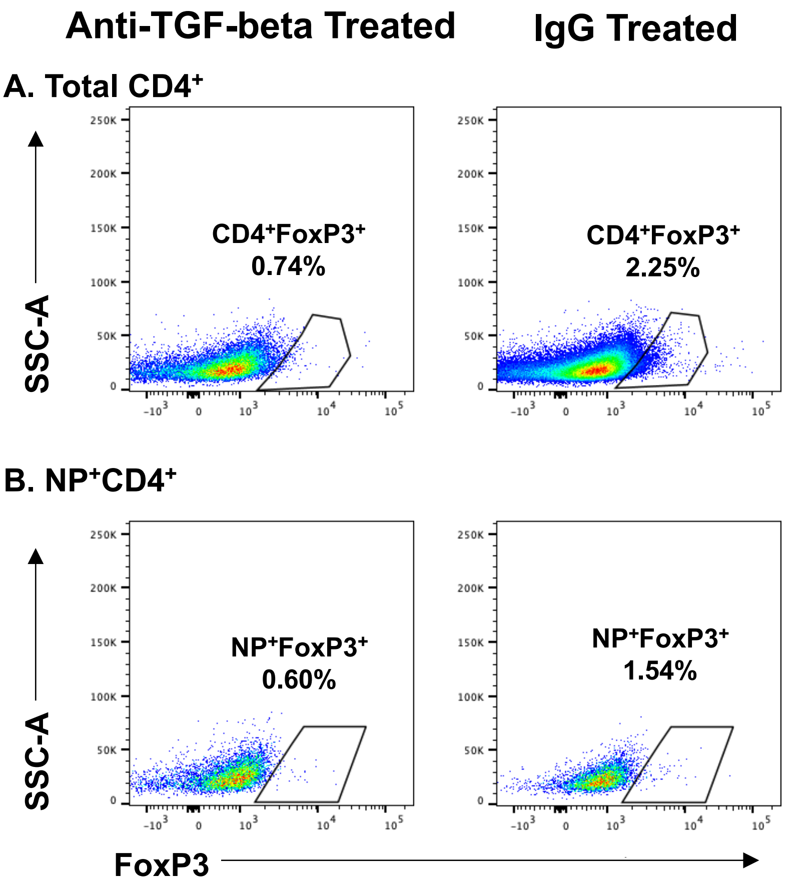

Supplemental figure 2. Flow cytometric analysis of CD4 T cells from anti-TGF-beta treated aged mice. Shows concatenated dot plots indicating the percent positive for FoxP3 and Tbet expression at the day 12 time point in Figure 2B (A. total CD4) and 2C (B. NP-specific CD4).
