# Supplementary material for: Senescence‐induced changes in CD4 T cell differentiation can be alleviated by treatment with senolytics
Source: Aging Cell. 2021 Dec 27;21(1):e13525. doi: 10.1111/acel.13525 (PMC8761018; doi:10.1111/acel.13525)
Supplement: Supplementary file 3 — Fig S3 [file ACEL-21-e13525-s002.pdf]

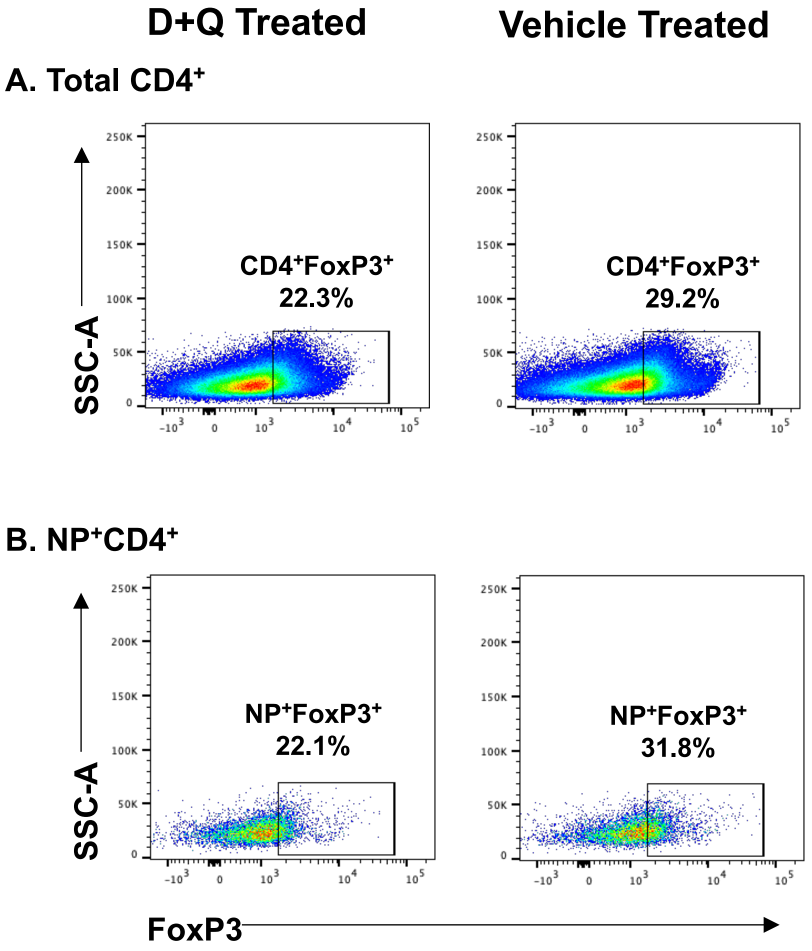

Supplemental figure 3. Flow cytometric analysis of CD4 T cells from D+Q treated aged mice. Shows concatenated dot plots indicating the percent positive for FoxP3 expression in: A. Total CD4 population and B. NP-specific CD4 population from Figure 3B.
