# Supplementary material for: Senescence‐induced changes in CD4 T cell differentiation can be alleviated by treatment with senolytics
Source: Aging Cell. 2021 Dec 27;21(1):e13525. doi: 10.1111/acel.13525 (PMC8761018; doi:10.1111/acel.13525)
Supplement: Supplementary file 4 — Fig S4 [file ACEL-21-e13525-s005.pdf]

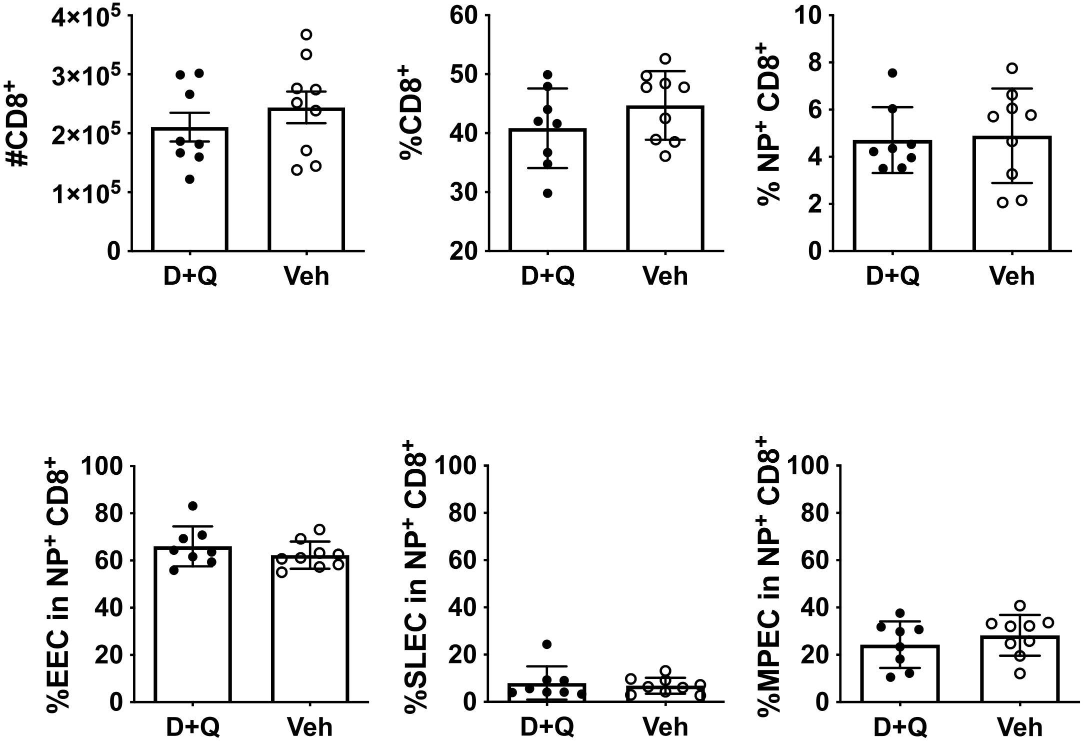

Supplemental figure 4. The impact of D+Q treatment on CD8 T cells in the lung during influenza infection. The experimental setup is shown in Figure 3. On day 12 post-infection, lymphocytes were recovered from the lungs. Flow cytometric analysis was used to identify the total CD8 T cell population and influenza NP-specific CD8 T cells (using H-2D^{b} influenza A NP$_{366-374}$ tetramer). CD8 T cell subsets were also identified: Early Effector Cells (EEC) KLRG-1^{lo} CD127^{lo}; Short Lived Effector Cells (SLEC) KLRG-1^{hi} CD127^{lo}; Memory Precursor Effector Cells (MPEC) KLRG-1^{lo} CD127^{hi}.
