# Supplementary material for: Senescence‐induced changes in CD4 T cell differentiation can be alleviated by treatment with senolytics
Source: Aging Cell. 2021 Dec 27;21(1):e13525. doi: 10.1111/acel.13525 (PMC8761018; doi:10.1111/acel.13525)
Supplement: Supplementary file 5 — Fig S5 [file ACEL-21-e13525-s004.pdf]

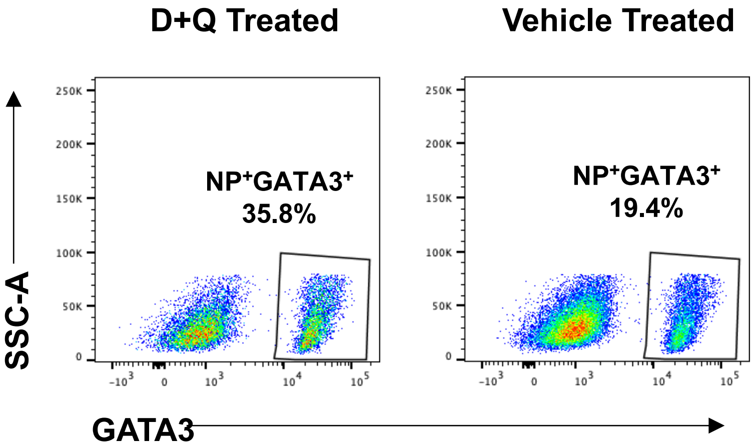

Supplemental figure 5. Flow cytometric analysis of NP-specific CD4 T cells from D+Q treated aged mice. Shows concatenated dot plots indicating the percent positive for GATA3 expression in the NP-specific CD4 population from Figure 4.
